# Supplementary material for: Macrophage-Specific, Mafb-Deficient Mice Showed Delayed Skin Wound Healing
Source: Int J Mol Sci. 2022 Aug 19;23(16):9346. doi: 10.3390/ijms23169346 (PMC9409077; doi:10.3390/ijms23169346)
Supplement: Supplementary file 1 [file ijms-23-09346-s001.zip › ijms-1866104-supplementary.pdf]

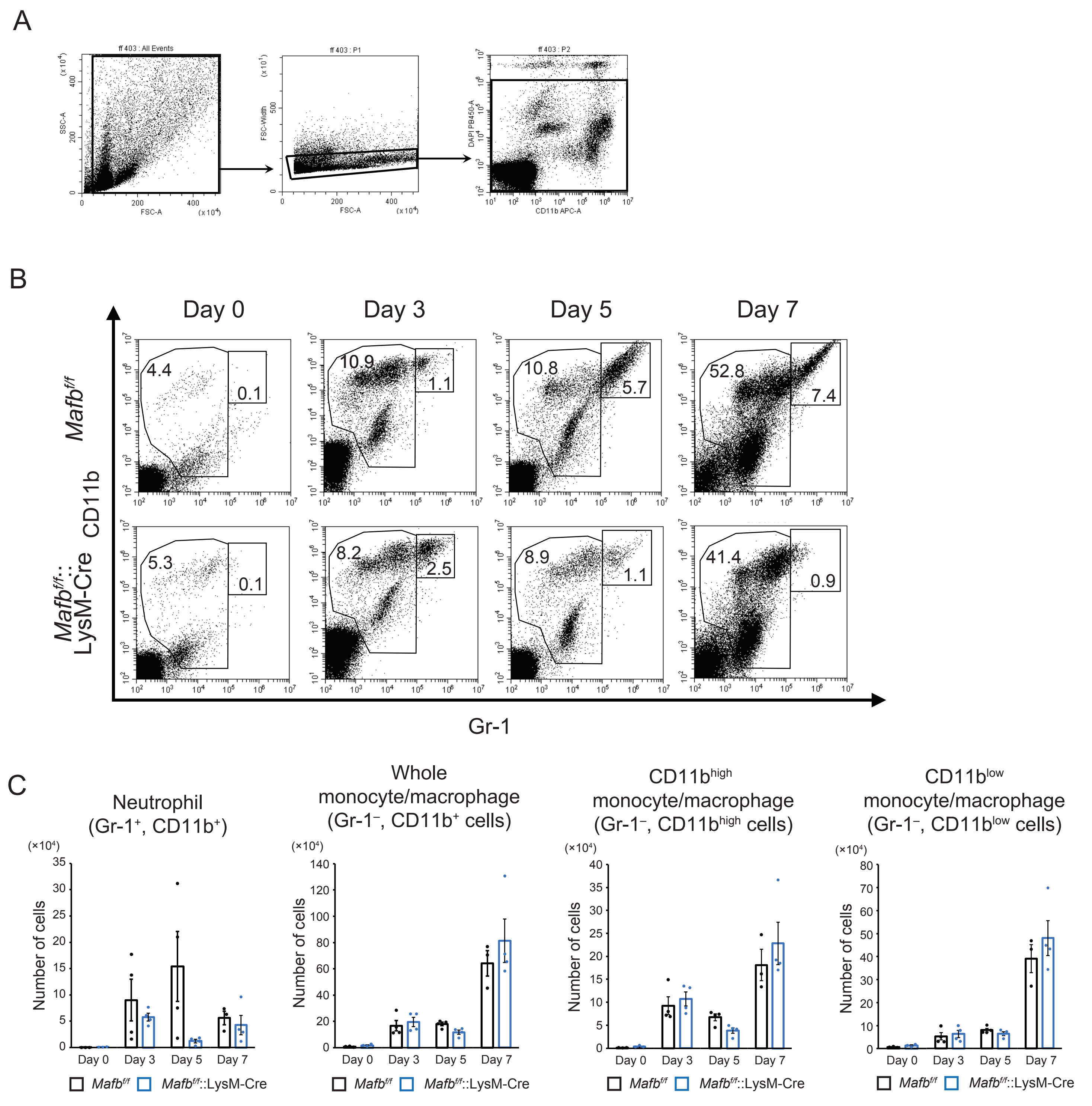

Suplemental Figure S1. The number of neutrophils and macrophages in wound

(A) Gating strategy of FACS analysis of wound tissue. (B) Cells in Day 0, 3, 5, and 7 wounds were stained with Gr-1 and CD11b antibodies and analyzed using flow cytometry. Dead cells were removed by DAPI staining shown as A. Representative dots plot was shown each day. (C) The absolute number of cells in each cell population shown in B was calculated from the overall number of cells taken from the tissue and the percentage of each cell population. Data are presented as the mean ± S.E.M; *p < 0.05, Student’ s t-test. Each Day used n=3-4 *Mafb*$^{f/f}$ and *Mafb*$^{f/f}$::LysM-Cre mice and each dot represent the data from one mouse.

A

Mafb$^{f/f}$ *Mafb*$^{f/f}$::LysM-Cre

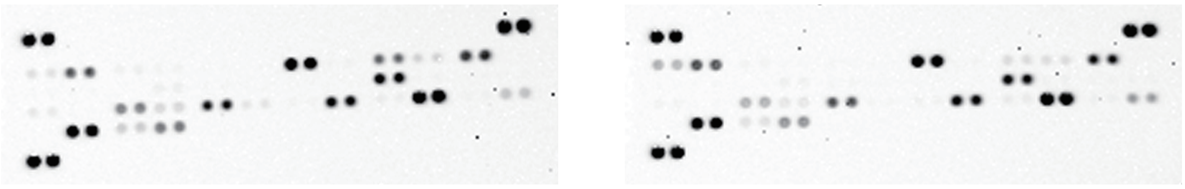

	1	2	3	4	5	6	7	8	9	10	11	12	13	14	15	16	17	18	19	20	21	22
A	Positive Control				ADAMTS1		Amphiregulin		Angiogenin		Angiopoietin-1		Angiopoietin-3		Coagulation Factor III		CXCL16				Positive Control	
B			IGFBP-10		DLL4		DPPIV		EGF		Endoglin		Collagen XVIII		Endothelin-1		FGF-1		FGF-2			
C			KGF		CX3CL1		GM-CSF		HB-EGF		Hepatopoietin A		IGFBP-1		IGFBP-2		IGFBP-3		IL-1α		IL-1β	
D			IL-10		CXCL10/CRG-2		CXCL1		Leption		CCL2		CCL3		MMP-3		MMP-8		MMP-9		CCN3/IGFBP-9	
E			Osteopontin		PD-ECGF		PDGF-AA		PDGF-AB/-BB		Pentraxin-3		CXCL4		PlGF-2		Prolactin		Proliferin			
F	Positive Control		CXCL12		Serpin E1		PEDF		Thrombospondin-2		TIMP-1		TIMP-4		VEGF		VEGF-B		Control (-)			

B

Mafb$^{f/f}$ *Mafb*$^{f/f}$::LysM-Cre

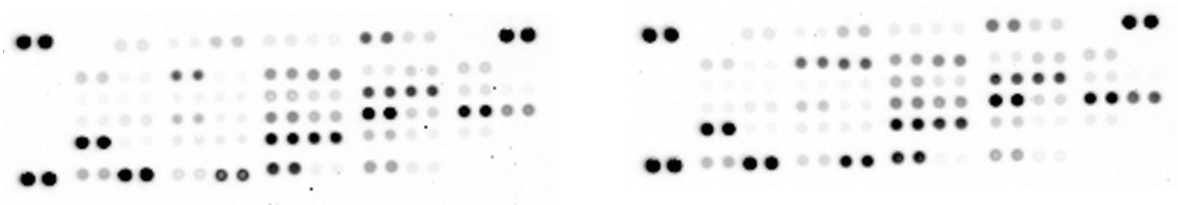

	1	2	3	4	5	6	7	8	9	10	11	12	13	14	15	16	17	18	19	20	21	22	23	24
A	Control																						Control	
B	CXCL13		C5/C5a		G-CSF		GM-CSF		CCL1		CCL11		sICAM-1		IFN-γ		IL-1α		IL-1β		IL-1ra		IL-2	
C	IL-3		IL-4		IL-5		IL-6		IL-7		IL-10		IL-13		IL-12 p70		IL-16		IL-17		IL-23		IL-27	
D	CXCL10		CXCL11		CXCL1		M-CSF		CCL2		CCL12		CXCL9		CCL3		CCL4		CXCL2		CCL5		CXCL12	
E	CCL17		TIMP-1		TNFα		TREM-1																	
F	Control																						Control (-)	

Suplemental Figure S2. Proteome analysis using Kits

(A) Inflammation and anti-inflammation cytokine array (*Mafb*$^{f/f}$, n = 2; *Mafb*$^{f/f}$::LysM-Cre, n = 2). (B) Angiogenesis-related protein array (*Mafb*$^{f/f}$, n = 4; *Mafb*$^{f/f}$::LysM-Cre, n = 4). The positions of the antibodies on each membrane are shown in a table. The vertical columns are shown in alphabetical order from A, starting from the top, and the horizontal rows are shown in numerical order from 1, starting from the left. Each protein was assessed in duplicate.

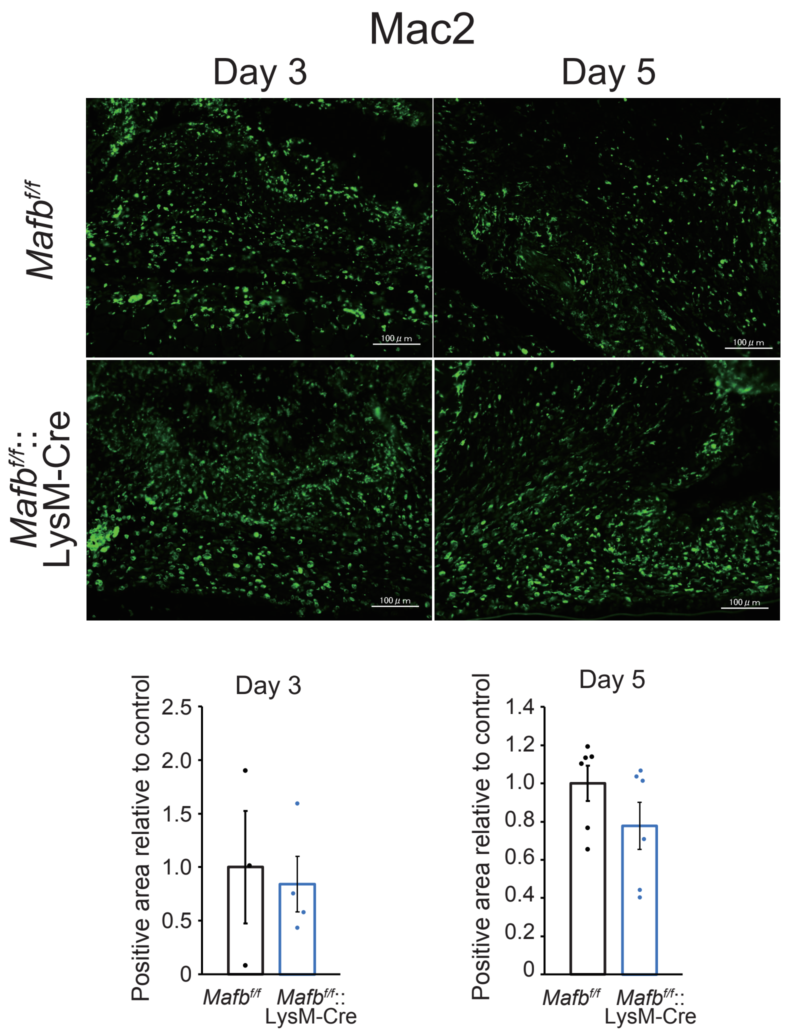

Suplemental Figure S3. Immunostaining of macrophages by Mac2

Immunostaining of Mac2 was performed in Day 3 and Day 5 wound tissue. The percentage of Mac2 positive area pre granulation tissue area was analyzed. Relative to control was shown in the graph.

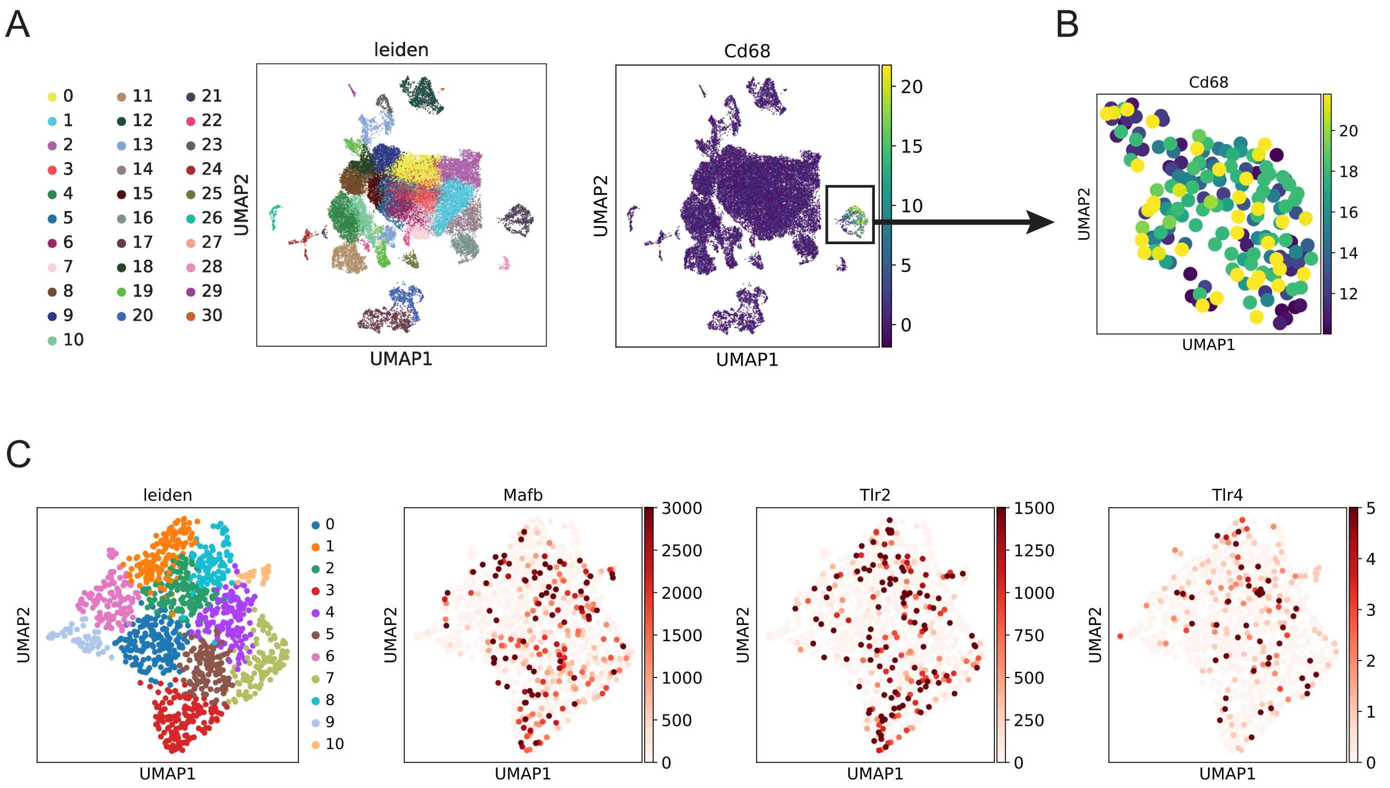

Suplemental Figure S4. The analysis of the scRNA-seq data

(A) Published scRNA-seq data of whole Day 5 wounds tissue were analyzed with Umap analysis. Cd68+ cells were shown. (B) The higher expression of Cd68 (the value over 10) were extracted. (C) Published scRNA-seq data of wound macrophages were analyzed with Umap analysis. The expression of *Mafb*, *Tlr2*, and *Tlr4* in wound macrophages were analyzed.

	Forward primer sequence (5'-3')	Reverse primer sequence (5'-3')
ArgI	TGGCTTGCGAGACGTAGAC	GCTCAGGTGAATCGGCCTTTT
CCL2	TGTTGGCTCAGCCAGATGCA	AGCCTACTCATTGGGATCATCTTG
CCL12	ACCATCAGTCCTCAGGTATTGG	TTCCGGACGTGAATCTTCTG
CXCL10	TGAATCCGGAATCTAAGACCATCAA	AGGACTAGCCATCCACTGGGTAAAG
EGF	TCATCTGCTCTAATGCAGGTACA	GTTTCCACAGTAACACTTCCCA
HPRT	CAAACTTTGCTTTCCCTGGT	CAAGGGCATATCCAACAACA
IL-10	CCAGGGAGATCCTTTGATGA	CATTCCCAGAGGAATTGCAT
IL-12b	GGAAGCACGGCAGCAGAATAA	CTTGAGGGAGAAGTAGGAATG
iNOS	ATGGCTTGCCCCTGGAAGTTTC	CAAGACTTGGACTTGCAAGTG
PRL	CTCAGGCCATCTTGGAGAAG	GAAGTGGGGCAGTCATTGAT

Table S1. Primer sequences used for RT-qPCR
